# Iron loading induces cholesterol synthesis and sensitizes endothelial cells to TNFα-mediated apoptosis

**DOI:** 10.1016/j.jbc.2021.101156

**Published:** 2021-09-02

**Authors:** Allison L. Fisher, Daniel N. Srole, Nicolaos J. Palaskas, David Meriwether, Srinivasa T. Reddy, Tomas Ganz, Elizabeta Nemeth

**Affiliations:** 1 Molecular, Cellular and Integrative Physiology Graduate Program, Graduate Programs in Bioscience, UCLA, Los Angeles, California, USA; 2 Department of Medicine, Center for Iron Disorders, David Geffen School of Medicine, UCLA, Los Angeles, California, USA; 3 Molecular and Medical Pharmacology Graduate Program, Graduate Programs in Bioscience, UCLA, Los Angeles, California, USA; 4 Division of Cardiology, Department of Medicine, UCLA, Los Angeles, California, USA

**Keywords:** iron metabolism, cholesterol metabolism, SREBP, tumor necrosis factor (TNF), TNFR1, apoptosis, caspase, lipid raft, FAC, ferric ammonium citrate, FAS, ferrous ammonium sulfate, HPCD, (2-hydroxypropyl)-β-cyclodextrin, HUVECs, human umbilical vein endothelial cells, MβCD, cholesterol-methyl-β-cyclodextrin, MCD, methyl-β-cyclodextrin, NTBI, non–transferrin-bound iron, SREBP, sterol regulatory element-binding protein, *TFRC*, transferrin receptor 1, TNFα, tumor necrosis factor-α

## Abstract

In plasma, iron is normally bound to transferrin, the principal protein in blood responsible for binding and transporting iron throughout the body. However, in conditions of iron overload when the iron-binding capacity of transferrin is exceeded, non–transferrin-bound iron (NTBI) appears in plasma. NTBI is taken up by hepatocytes and other parenchymal cells *via* NTBI transporters and can cause cellular damage by promoting the generation of reactive oxygen species. However, how NTBI affects endothelial cells, the most proximal cell type exposed to circulating NTBI, has not been explored. We modeled *in vitro* the effects of systemic iron overload on endothelial cells by treating primary human umbilical vein endothelial cells (HUVECs) with NTBI (ferric ammonium citrate [FAC]). We showed by RNA-Seq that iron loading alters lipid homeostasis in HUVECs by inducing sterol regulatory element-binding protein 2–mediated cholesterol biosynthesis. We also determined that FAC increased the susceptibility of HUVECs to apoptosis induced by tumor necrosis factor-α (TNFα). Moreover, we showed that cholesterol biosynthesis contributes to iron-potentiated apoptosis. Treating HUVECs with a cholesterol chelator hydroxypropyl-β-cyclodextrin demonstrated that depletion of cholesterol was sufficient to rescue HUVECs from TNFα-induced apoptosis, even in the presence of FAC. Finally, we showed that FAC or cholesterol treatment modulated the TNFα pathway by inducing novel proteolytic processing of TNFR1 to a short isoform that localizes to lipid rafts. Our study raises the possibility that iron-mediated toxicity in human iron overload disorders is at least in part dependent on alterations in cholesterol metabolism in endothelial cells, increasing their susceptibility to apoptosis.

Iron is an essential micronutrient but can cause tissue damage when in excess. In healthy humans and animals, iron in plasma is bound to the protein transferrin. When the iron-carrying capacity of transferrin is exceeded, such as in iron overload conditions, iron appears in plasma complexed with low-molecular-weight molecules, collectively referred to as non–transferrin-bound iron (NTBI) (1), with ferric citrate reported to be the predominant NTBI species (2, 3). NTBI is thought to contribute to organ dysfunction in iron overload conditions through the generation of reactive oxygen species that damage cellular lipids, proteins, and DNA (4) and can also promote susceptibility to certain infections (5). Because humans lack compensatory mechanisms to excrete excess iron, iron absorption is tightly regulated by the hormone hepcidin, which is produced in the liver. Hepcidin acts by binding to its receptor and only known iron exporter, ferroportin, blocking iron transport into plasma, and lowering plasma iron levels (6). Hepcidin deficiency results in iron overload in patients with hereditary iron disorders or ineffective erythropoiesis. Iron overload can also develop in people after repeated blood transfusions, or excessive iron supplementation.

NTBI accumulation in cells leads to their dysfunction, with specific tissue toxicities dependent on both the rate and extent of NTBI accumulation. The liver is not only the main storage organ for iron but also the organ most commonly damaged by chronic iron overload. Iron uptake pathways in hepatocytes include the classical transferrin–transferrin receptor–mediated uptake, as well as the uptake of NTBI through specific transporters. Hepatocytes take up NTBI at a rapid rate that exceeds their capacity for iron export, resulting in the net accumulation of excess iron when NTBI is chronically elevated (7). Although most NTBI in plasma is cleared by the liver, NTBI can also be taken up by the pancreas, kidney, and heart (8). How different cell types take up NTBI is an area of active investigation (9), but ZIP14 was shown to be the NTBI transporter in hepatocytes and pancreatic acinar cells (10, 11).

In patients with iron overload, circulating NTBI would come in contact with endothelial cells first, before reaching other cell types. However, the direct effects of NTBI on endothelial cells within the liver or other organs that accumulate iron in iron overload disorders are not fully understood. Of the different types of endothelial cells, those in the liver have emerged as a cell type with essential roles in iron homeostasis (12, 13). In response to liver iron loading, endothelial cells produce bone morphogenetic proteins, at least in part through oxidative stress–induced NRF2 transcription factor (14), and bone morphogenetic proteins exert paracrine effects on hepatocytes to induce hepcidin production and modulate systemic iron homeostasis (13). Whether iron has regulatory and/or toxic effects on endothelial cells in other organs remains to be determined.

In this study, we explored how NTBI accumulation, mimicking human iron overload, affects cultured primary human umbilical vein endothelial cells (HUVECs). Using unbiased RNA-Seq, we determined that iron loading of HUVECs by ferric ammonium citrate (FAC) potently induced lipid biosynthesis through the sterol regulatory element-binding protein (SREBP) pathways, predominantly affecting SREBP2-mediated cholesterol metabolism. We further found that cellular iron loading sensitized HUVECs to apoptotic cell death induced by tumor necrosis factor-α (TNFα) and that iron loading altered the TNFα pathway by generating a short isoform of TNFR1 through a novel proteolytic cleavage. Evaluating the contribution of cholesterol biosynthesis and TNFR1 cleavage to iron-potentiated apoptosis, we show that pharmacological depletion of cholesterol was sufficient to rescue the apoptotic phenotype even in the presence of excess iron and the presence of the short TNFR1 isoform. Thus, we provide specific evidence that iron loading alters endothelial cell cholesterol homeostasis and increases their susceptibility to inflammation-mediated apoptosis. Our findings could have pathophysiological relevance for patients with iron-loading disorders affected by acute or chronic inflammation.

## Results

### Iron induces cholesterol biosynthesis

**Figure 1:**
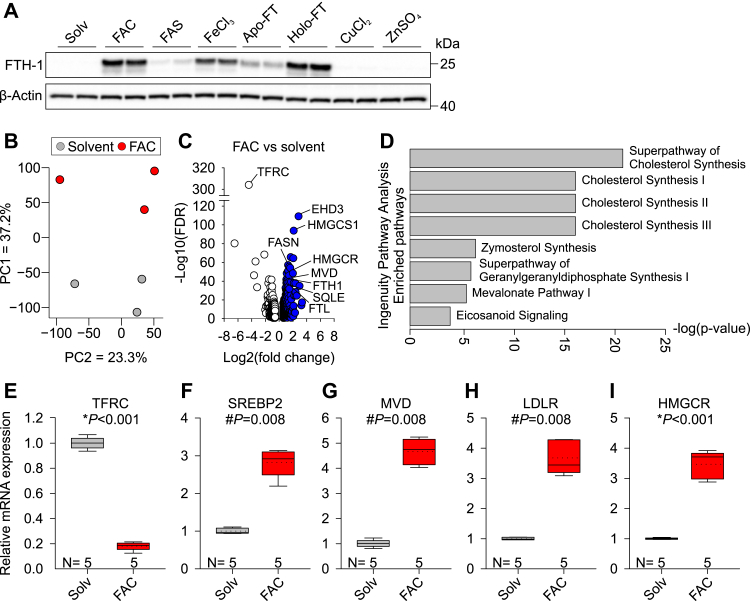
**Iron induces cholesterol biosynthesis in primary HUVECs.***A*, Western blot for iron storage protein FTH1 in HUVECs treated with solvent (water) or 100 μM FAC, ferric ammonium sulfate (FAS, 100 μM), ferric chloride (FeCl_3_, 100 μM), apoferritin (FT, 2 mg/ml), holo-FT (2 mg/ml), copper chloride (CuCl_2_, 100 μM), or zinc sulfate (ZnSO_4_, 100 μM) for 24 h. β-Actin was used as a loading control. *B*–*D*, to induce cellular iron loading, primary HUVECs were treated with solvent (water) or 100 μM FAC for 30 h, and total RNA was collected for RNA-Seq. *B*, principal component scatter plot of gene expression in FAC-treated (*red circles*) *versus* solvent-treated (*gray circles*) HUVECs. The percentages of each axis represent the percentage of variation explained by the principal components. *C*, volcano plot of 816 differentially expressed genes comparing FAC- *versus* solvent-treated HUVECs. Differentially expressed genes were identified by fold change of greater or less than ±1.5 and *p* < 0.05. *White circles* represent downregulated genes, and *blue circles* represent upregulated genes. *D*, ingenuity pathway analysis identifying significantly enriched categories between FAC- and solvent-treated HUVECs (top hits shown, based on *p* < 0.001 and *z*-score > 2). *E*–*I*, primary HUVECs were treated with solvent (water, *gray*) or 100 μM FAC (*red*) for 40 h. qPCR analysis of (*E*) iron importer *TFRC* and (*F*–*I*) cholesterol biosynthesis genes *SREBP2*, *MVD*, *LDLR*, and *HMGCR*. Data are shown as 2^−ΔΔCt^. The number of biological replicates is indicated above the *x*-axis. Statistical differences between groups were determined by Student’s *t* test for normally distributed values (denoted by ∗) or Mann–Whitney *U* for non-normally distributed values (denoted by #). FAC, ferric ammonium citrate; HUVECs, human umbilical vein endothelial cells.

We used a cell culture system to model the effects of systemic iron overload on endothelial cells by treating primary HUVECs with NTBI in the form of FAC, a form of iron that rapidly and effectively loaded HUVECs without causing toxicity (Fig. 1*A*). We chose prolonged exposures (30–40 h of NTBI) based on preliminary experiments assessing HUVECs rate of iron loading and evidence of cellular damage. To identify global transcriptome changes in endothelial cells in response to NTBI, we performed RNA-Seq analysis of HUVECs treated with 100 μM FAC for 30 h. Principal component analysis of RNA-Seq data showed that solvent- and FAC-treated groups were in distinct clusters and indicated that the first principal component explains almost 40% of the variability among samples (Fig. 1*B*). Volcano plot showed 816 differentially expressed genes, including both downregulated and upregulated genes, between solvent and FAC-treated HUVECs (Fig. 1*C*). Transferrin receptor 1 (*TFRC*) mRNA levels, which are regulated by cellular iron loading *via* a post-transcriptional mechanism (15), were significantly decreased with FAC treatment (Fig. 1*C*), confirming that cells were effectively iron loaded. Ingenuity pathway analysis of FAC- and solvent-treated cells revealed that FAC primarily stimulates cholesterol biosynthesis pathways (Fig. 1*D*). We validated the RNA-Seq findings by measuring mRNA expression of genes involved in cholesterol biosynthesis by qRT-PCR in a separate set of HUVECs, treated with 100 μM FAC for 40 h. The cells were effectively iron-loaded, as reflected by the decrease in *TFRC* mRNA expression (*p* < 0.001, *t* test, Fig. 1*E*). We confirmed increased expression of *SREBP2*, a transcription factor that is a master regulator of cholesterol synthesis (*p* = 0.008, Mann–Whitney *U*). The expression of SREBP2 target genes was likewise upregulated: mevalonate diphosphate decarboxylase (*MVD*) (*p* = 0.008, Mann–Whitney *U*), low-density lipoprotein receptor (*LDLR*) (*p* = 0.008, Mann-Whitney *U*), and 3-hydroxy-3-methylglutaryl-CoA reductase (*HMGCR*) (*p* < 0.001, *t* test) (Fig. 1, *F*–*I*). Furthermore, we measured *SREBP2* mRNA expression in HUVECs treated with different forms of iron—NTBI (FAC, ferrous ammonium sulfate [FAS], ferric chloride), transferrin-bound iron, holo-ferritin and hemin—or with triammonium citrate as a control (Fig. S1*A*). Most of the iron treatments (except for FAS) induced *SREBP2* mRNA, whereas triammonium citrate (lacking iron) did not. Expression of *SREBP2* correlated with the degree of cellular iron loading as measured by ferritin heavy chain immunoblotting (r = 0.826, *p* = 0.012, Fig. S1*B*). The lack of *SREBP2* induction in the FAS condition is presumably related to inefficient cellular iron loading with FAS treatment. This result demonstrated that it is the cellular iron loading, as opposed to the specific form of NTBI, that induces cholesterol synthesis.

We further measured mRNA expression of SREBP transcription factors that control fatty acid biosynthetic pathways. FAC treatment of HUVECs increased expression of *SREBP1* isoforms a and c (both *p* < 0.001, *t* test) and their gene targets acetyl-CoA carboxylase (*ACCA*) (*p* = 0.002, *t* test), and fatty acid synthase (*FASN*) (*p* < 0.001, *t* test) (Fig. S2, *A*–*E*), suggesting that iron plays a broader role in lipid homeostasis. Taken together, these findings demonstrate that iron loading of endothelial cells induces expression of the SREBP pathway genes that control sterol and fatty acid synthesis.

**Figure 2:**
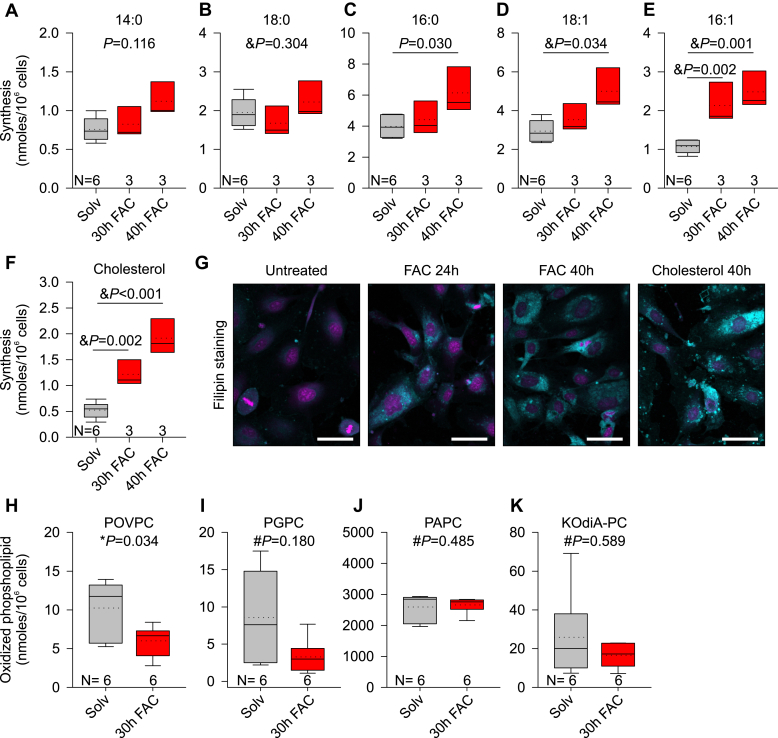
**Lipid composition in control and iron-loaded HUVECs.***A*–*F*, isotopic spectral analysis of HUVECs treated with solvent (water, *gray*) or 100 μM FAC (*red*) for 30 or 40 h in the presence of 5 mM [U^13^C]-D-glucose. Lipid synthesis is expressed as nanomoles/million cells: cellular (*A*) myristic acid (14:0), (*B*) stearic acid (18:0), (*C*) palmitic acid (16:0), (*D*) oleic acid (18:1), (*E*) palmitoleic acid (16:1), and (*F*) cholesterol. *G*, confocal microscopy of filipin fluorescence staining of cholesterol (*turquoise* pseudo color) of HUVECs treated with 100 μM FAC for 24 or 40 h. HUVECs treated with 50 μM cholesterol-methyl-β-cyclodextrin (MβCD) were used as a positive control. Nuclear stain (*violet* pseudo color) = SYTOX green. The scale bar represents 50 μm. *H*–*K*, LC-MS analysis of oxidized phospholipids in HUVECs treated with solvent (water) or 100 μM FAC for 30 h. *H*, 1-palmitoyl-2-(5-oxovaleroyl)-sn-glycero-3-phosphocholine (POVPC), (*I*) 1-palmitoyl-2-glutaroyl-sn-glycero-3-phosphocholine (PGPC), (*J*) 1-palmitoyl-2-arachidonoyl-sn-glycero-3-phosphocholine (PAPC), and (*K*) 1-(palmitoyl)-2-(5-keto-6-octene-dioyl)phosphatidylcholine (KOdiA-PC). The number of replicates is indicated above the *x*-axis. Statistical differences between groups were determined by one-way ANOVA on ranks with Dunn’s method of multiple comparison for non-normally distributed values, one-way ANOVA followed by Holm–Sidak method of multiple comparisons for normally distributed values (denoted by &), Student’s *t* test (denoted by ∗), or Mann–Whitney *U* for non-normally distributed values (denoted by #). FAC, ferric ammonium citrate; HUVECs, human umbilical vein endothelial cells.

To assess the effect on lipid synthesis, HUVECs were labeled with [U^13^C]-D-glucose and treated with FAC for 30 and 40 h, the two time points used for transcriptional analysis in Figure 1. Using isotopomer spectral analysis, we did not detect any changes in the synthesis of saturated fatty acids myristic acid (14:0) or stearic acid (18:0) with FAC treatment (Fig. 2, *A* and *B*). FAC treatment mildly increased synthesis of palmitic acid (16:0) (*p* = 0.030, one-way ANOVA) and oleic acid (18:1) (*p* = 0.034, one-way ANOVA) by 1.5-fold after 40 h (Fig. 2, *C* and *D*), and more strongly induced synthesis of unsaturated fatty acid palmitoleic acid (16:1) by >2-fold at both time points (*p* = 0.002 at 30 h, and *p* < 0.001 at 40 h, one-way ANOVA) (Fig. 2*E*). FAC had the strongest effect on cholesterol biosynthesis, with 4-fold induction achieved by 40 h (Fig. 2*F*; *p* = 0.002 at 30 h and *p* < 0.001 at 40 h, one-way ANOVA). Increases in cellular cholesterol were also confirmed by staining of HUVECs with the fluorescent cholesterol probe filipin after FAC treatment (Fig. 2*G*). Cholesterol-treated HUVECs were used as a control for filipin staining.

Iron can damage cellular membrane integrity by generating hydroxyl radicals through the Fenton reaction that cause peroxidation of membrane phospholipids (4). We considered whether cellular membrane damage mediates the induction in cholesterol biosynthesis by determining whether FAC caused peroxidation of membrane phospholipids. We measured 1-palmitoyl-2-(5-oxovaleroyl)-sn-glycero-3-phosphocholine, 1-palmitoyl-2-glutaroyl-sn-glycero-3-phosphocholine, and 1-(palmitoyl)-2-(5-keto-6-octene-dioyl)phosphatidylcholine, which are common stable end-products of and biomarkers for lipid peroxidation of palmitoyl arachidonoyl phosphatidylcholine (Fig. 2, *H*–*K*) (16). We did not detect any major iron-dependent changes in the cellular levels of these phospholipid peroxidation markers. These data indicate that FAC did not damage the cell membranes through peroxidation and suggests that iron stimulates cholesterol biosynthesis independently of membrane damage.

To ascertain whether iron loading of other cell types alters their *SREBP2* mRNA, human cell lines Hep3B (hepatocellular carcinoma) and immortalized endothelial line teloHAEC were treated with 100 μM FAC for 40 h. FAC decreased *TFRC* expression in both Hep3B and teloHAEC, confirming that these cell lines efficiently load FAC (Fig. S3*A*). Despite this, neither Hep3B nor teloHAEC induced *SREBP2* expression, and Hep3Bs even moderately reduced *SREBP2* expression (Fig. S3*B*). This result suggests that although iron-dependent changes in cholesterol homeostasis occur in primary endothelial cells, such a cellular response to iron loading is not universal.

### Cellular iron loading potentiates apoptosis

**Figure 3:**
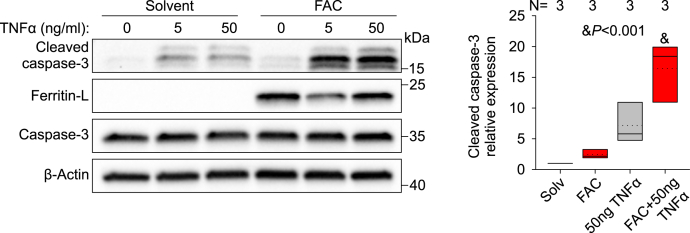
**Iron loading of HUVECs sensitizes them to apoptosis.** Primary HUVECs were treated with solvent (water, *gray*) or 100 μM FAC (*red*) for 24 h before stimulation with 5 or 50 ng/ml TNFα in normal or FAC-supplemented media for 6 h. Western blot for cleaved caspase-3. Representative image of N = 3 independent experiments. Quantification of cleaved caspase-3 normalized to β-actin is shown for the 50 ng/ml dose. Statistical differences between groups were determined by one-way ANOVA for normally distributed values. HUVECs, human umbilical vein endothelial cells; TNFα, tumor necrosis factor-α.

In iron overload conditions, NTBI causes toxicity through promoting the formation of free radicals in tissues in which it accumulates. We next evaluated the sensitivity of endothelial cells to iron-mediated cytotoxicity. Caspase-3 is known to be a critical executioner of apoptosis, and its activation requires proteolytic processing of the proenzyme (17, 18). We therefore determined the levels of cleaved caspase-3 as a marker of apoptosis in HUVECs treated with 100 μM FAC for 30 h, but FAC treatment alone did not cause apoptosis (Fig. 3). We then assessed the effect of iron loading on apoptosis triggered by TNFα, a canonical apoptotic stimulus. TNFα treatment (5 and 50 ng/ml for 6 h) by itself mildly increased cleaved caspase-3. However, we observed a strong interaction between iron and the inflammatory cytokine, where FAC treatment significantly potentiated cleaved caspase-3 induced by TNFα (Fig. 3). The result indicates that iron loading increases the susceptibility of endothelial cells to inflammatory damage. We did not observe any FAC-dependent potentiation of cleaved caspase-3 by TNFα in immortalized Hep3B or teloHAEC (Fig. S4), suggesting that primary endothelial cells are unusually susceptible to iron-potentiated apoptosis.

### Iron modulates TNFR1 proteolytic processing

**Figure 4:**
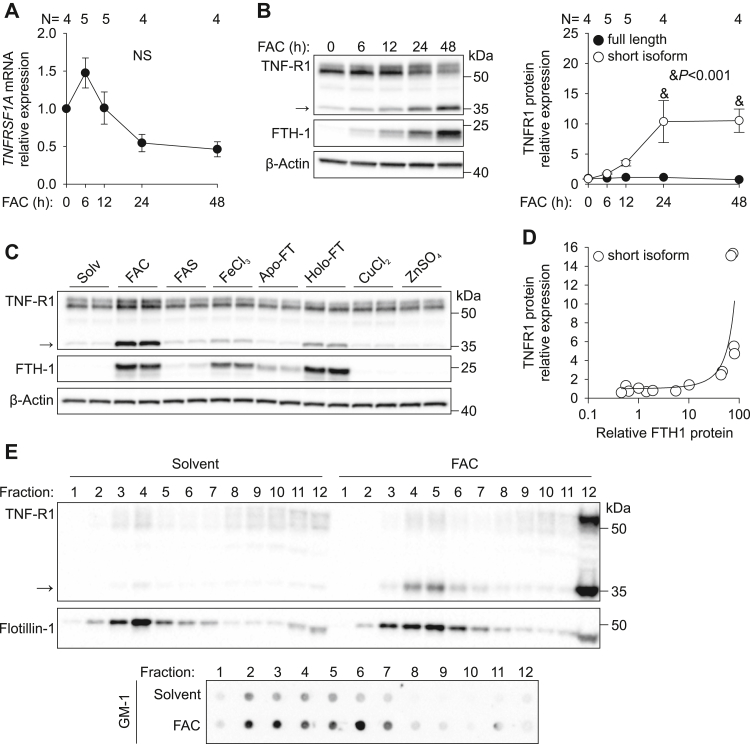
**In HUVECs, iron loading induces expression of a short isoform of TNFR1 that localizes to lipid rafts.***A*, primary HUVECs were treated with 100 μM FAC for 0 to 48 h. *TNFRSF1A* mRNA analysis by qRT-PCR normalized to *HPRT* and expressed as 2^−ΔΔCt^. *B*, Western blot and protein quantification of the full-length (55 kDa) and short TNFR1 isoform (35 kDa, indicated by an *arrow*) normalized to β-actin. *C*, Western blot for TNFR1 in HUVECs treated with solvent (water) or 100 μM FAC, ferric ammonium sulfate (FAS, 100 μM), ferric chloride (FeCl_3_, 100 μM), apoferritin (FT, 2 mg/ml), holo-FT (2 mg/ml), copper chloride (CuCl_2_, 100 μM), or zinc sulfate (ZnSO_4_, 100 μM) for 24 h. The FTH1 and β-actin blots are replicated from Figure 1*A* but are provided here for clarity. *D*, correlation between FTH1 and short TNFR1 isoform normalized to β-actin. *E*, HUVECs treated with solvent (water) or 100 μM FAC for 24 h were subjected to sucrose density gradient centrifugation to isolate lipid rafts. Proteins from equal volume of collected fractions were separated by SDS-PAGE and analyzed by Western blotting. To analyze the distribution of GM-1, each fraction was dot-blotted onto a nitrocellulose membrane and detected using CTx^HRP^. Statistical differences were determined by one-way ANOVA with the Holm–Sidak method of multiple comparisons. FAC, ferric ammonium citrate; HUVECs, human umbilical vein endothelial cells; NS, not significant.

We next evaluated whether iron alters signaling downstream of TNFα, which could increase susceptibility to cell death. TNFα has two main receptors, TNFR1 (or p55) and TNFR2 (or p75), which are expressed on many cell types but belong to different subgroups of the TNFR family. TNFR1 is a death receptor and harbors a death domain in its cytoplasmic portion, which links TNFR1 to apoptosis and necroptosis (19). In comparison, TNFR2 does not contain a death domain. TNFR1 is a 55-kDa transmembrane protein that is shed into circulation as 27- to 30-kDa soluble proteins after extracellular cleavage (20, 21), and the soluble form can inhibit TNFα by competing for TNFα binding. We therefore evaluated the effect of excess iron on TNFR1 expression. HUVECs were treated with FAC over a time course between 0 and 48 h, and we assessed TNFR1 mRNA and protein expression. FAC treatment did not significantly alter *TNFRSF1A* mRNA expression (Fig. 4*A*). However, FAC treatment strongly affected TNFR1 protein composition. Although the longest isoform (55-kDa) was relatively stable with FAC treatment, a novel shorter isoform (35-kDa) was induced 10-fold at 24 and 48 h (*p* < 0.001, one-way ANOVA) (Fig. 4*B*). To ensure that the 35-kDa isoform is TNFR1 rather than a result of nonspecific antibody interaction with another protein, we validated the finding by depleting HUVECs of endogenous TNFR1 using control siRNA (siNC) or siRNA targeting TNFR1 (siTNFR1) for 48 h and treating the cells with FAC or solvent for 24 h. Treatment with siTNFR1 reduced the mRNA levels ∼5-fold and reduced protein expression of both the full-length and shorter isoform of TNFR1 (Fig. S5, *A* and *B*), confirming the specificity of the antibody.

We next tested whether the induction of the smaller 35-kD isoform was specifically caused by cellular iron loading. We treated HUVECs with FAC, FAS, ferric chloride (FeCl_3_), apo-ferritin (apo-FT, ferritin lacking iron), holo-ferritin (holo-FT, ferritin containing iron), copper chloride (CuCl_2_), or zinc sulfate (ZnSO_4_) for 24 h. Induction of the short TNFR1 isoform was strongest with FAC treatment, occurred to a lesser extent with FeCl_3_ and holo-FT, and was not induced by FAS, apo-FT, copper, or zinc, which did not cause iron loading (Fig. 4*C*). In agreement, the levels of the short TNFR1 isoform correlated with the degree of iron loading as measured by expression of iron storage protein ferritin heavy chain (FTH1), with an apparent threshold at FTH1/β-actin of about 30 (Fig. 4*D*), whereas the levels of the full-length isoform did not correlate with FTH1 expression (Fig. S6).

To determine the cellular localization of the short TNFR1 isoform, we performed membrane enrichment assays. In view of the reported recruitment of TNFR1 to lipid rafts (22), we determined the localization of the short TNFR1 isoform in membrane fractions from HUVECs treated with the solvent or FAC for 24 h. Using density-gradient centrifugation, we found that FAC treatment upregulates expression of the short form of TNFR1 in the lipid raft fractions, as defined by the enrichment of flotillin-1 and ganglioside GM-1 (Fig. 4*E*).

**Figure 5:**
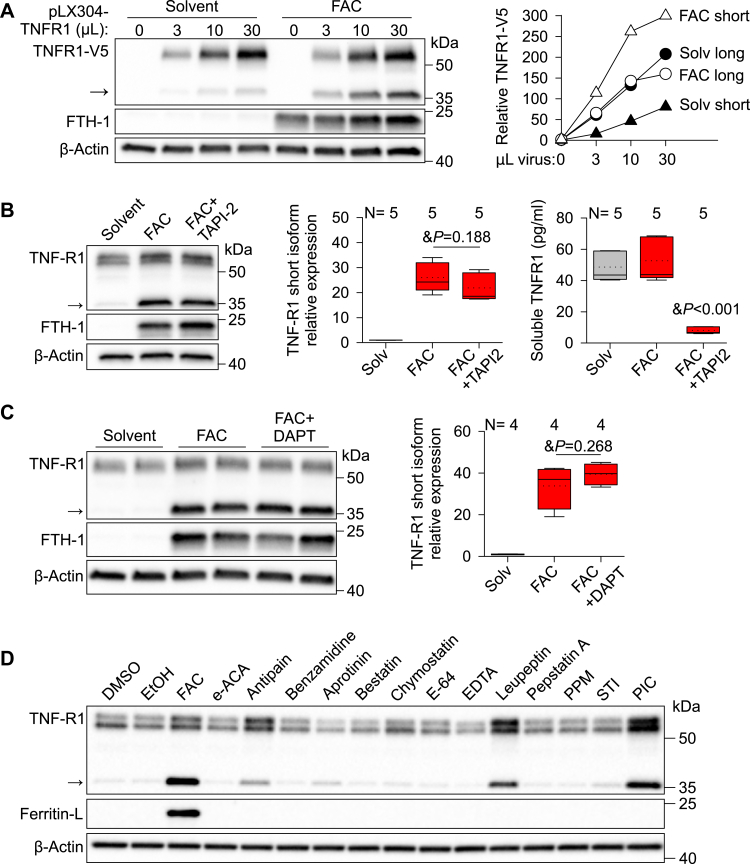
**Iron induces post-translational modifications of TNFR1.***A*, HUVECs were transduced with increasing volume of lentivirus expressing human TNFR1 with a C-terminal V5 tag for 10 h before incubation with solvent (water) or 100 μM ferric ammonium citrate (FAC) for 24 h. Western blot using anti-V5 antibody; the short isoform indicated by an *arrow*. *B*, HUVECs were treated with solvent (water, *gray*) or 100 μM FAC (*red*) with and without 25 μM TAPI-2 inhibitor for 24 h and Western blot performed for TNFR1. Representative image showing endogenous TNFR1 (*left*), quantification of the small TNFR1 isoform (*middle*), and quantification of soluble TNFR1 in HUVEC supernatants (*right*). *C*, Western blot and quantification of small TNFR1 isoform after HUVECs were treated with solvent (water) or 100 μM FAC with and without 1 μM DAPT inhibitor. *D*, Western blot for TNFR1 in HUVECs treated with solvents DMSO or ethanol, or a panel of protease inhibitors: 6-aminohexanoic acid (E-ACA, 40 mM), antipain (100 μM), benzamidine HCl (4 mM), aprotinin (1 μM), bestatin (40 μM), chymostatin (100 μM), E-64 (10 μM), EDTA (1 mM), leupeptin (100 μM), pepstatin A (1 μM), phosphoramidon (PPM, 10 μM), soybean trypsin inhibitor (STI, 5 μM), or protease inhibitor cocktail (PIC, 1:200) for 24 h. FAC was used as a positive control. β-Actin was used as a loading control. The number of replicates is indicated above the *x*-axis. Statistical differences between groups were determined by one-way ANOVA with the Holm–Sidak method for multiple comparisons for normally distributed values. FAC, ferric ammonium citrate; HUVECs, human umbilical vein endothelial cells.

We next examined whether iron induces proteolytic processing of TNFR1 to generate the shorter isoform. HUVECs were transduced with lentivirus expressing TNFR1 (pLX304-TNFR1) before FAC treatment. Because the lentivirus contains TNFR1 cDNA, any change in TNFR1 would be from post-translational modifications rather than generation of new mRNA isoforms. FAC treatment of HUVECs overexpressing TNFR1 resulted in a strong induction in the short but not full-length TNFR1 isoform (Fig. 5*A*), confirming that TNFR1 regulation by iron is a result of proteolytic processing rather than generation of a new mRNA isoform. Considering this, we first evaluated the role of the canonical TNFR1 cleavage pathways in the induction of the short TNFR1 isoform. These pathways have been previously shown to generate a 27- to 30-kDa soluble fragment (23) and 26- to 30-kDa cell-associated fragment of TNFR1 (20, 24). Treatment of FAC-loaded HUVECs with TAPI2, an ADAM17 (TNFα converting enzyme), and matrix metalloprotease inhibitor in combination with FAC did not change the amount of the lower TNFR1 isoform, despite a reduction in soluble TNFR1 in supernatants (Fig. 5*B*). Furthermore, treatment of HUVECs with DAPT, a γ-secretase inhibitor, before iron loading did not alter expression of the short TNFR1 isoform (Fig. 5*C*), suggesting that generation of the 35-kDa isoform is independent of the canonical TNFR1 cleavage processes. Further evaluation of proteolytic processing using a panel of protease inhibitors in HUVECs showed that protease inhibitors antipain (serine inhibitor) and leupeptin (serine & thiol inhibitor) increased expression of the short TNFR1 isoform similarly as FAC treatment or treatment with a protease inhibitor cocktail (containing aprotinin, bestatin, E-64, leupeptin, and pepstatin A) (Fig. 5*D*), demonstrating that FAC treatment mimics the action of protease inhibitors and thereby suggesting that FAC may antagonize or inactivate one or more serine proteases.

In addition to the known *N*-linked glycosylation sites on TNFR1 (asparagine 54, 145, and 151), we considered whether the 35-kDa isoform may be a result of glycosylation of an even smaller fragment, by treating HUVECs with FAC for 30 h and evaluating *N*- and *O*-linked glycosylation. As expected, the full-length TNFR1 isoform was *N*-linked glycosylated, as treatment with PNGaseF resulted in a downward shift in the molecular weight of full-length TNFR1 compared with untreated and *O*-glycosidase–treated lysates (Fig. S7). However, the molecular weight of the short TNFR1 isoform was similar between untreated and *N*- and *O*-deglycosylated lysates (Fig. S7), indicating that the short isoform is not glycosylated. We similarly detected induction of the short TNFR1 isoform with FAC loading in Hep3B and teloHAEC (Fig. S8), suggesting a more common role of iron in TNFR1 processing.

### Cellular cholesterol loading potentiates apoptosis and promotes TNFR1 processing

**Figure 6:**
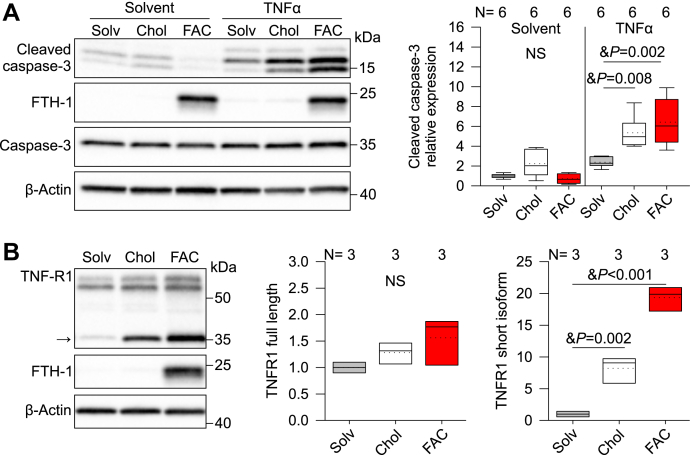
**Cholesterol treatment potentiates apoptosis and increases TNFR1 short isoform in HUVECs.***A*, cleaved caspase-3 Western blot of HUVECs treated with solvent (water, *gray*), 50 μM cholesterol-MβCD (*white*), or 100 μM FAC (*red*) followed by stimulation with 50 ng/ml TNFα for 16 h. *B*, Western blot and protein quantification of full-length and TNFR1 short isoform (indicated by an *arrow*) in HUVECs treated with solvent (water, *gray*), 50 μM cholesterol-MβCD (*white*), or 100 μM FAC (*red*) for 40 h. Representative images of N = 3 independent experiments. β-Actin was used as a loading control. Statistical differences between groups were determined by one-way ANOVA with the Holm–Sidak method for multiple comparisons for normally distributed values. FAC, ferric ammonium citrate; HUVECs, human umbilical vein endothelial cells; MβCD, cholesterol-methyl-β-cyclodextrin.

Our data show that iron alters lipid homeostasis and proteolytic processing of TNFR1 in endothelial cells. We next asked whether altered lipid homeostasis or TNFR1 expression contributes to iron potentiation of apoptosis. We tested the contribution of high cellular cholesterol on apoptosis by treating HUVECs with 50 μM cholesterol-methyl-β-cyclodextrin (MβCD) for 24 h before stimulation with 50 ng/ml TNFα for 16 h and compared the apoptotic response seen with the combination of FAC and TNFα. Cholesterol-MβCD by itself had no effect on cleaved caspase-3, but cotreatment with TNFα did potentiate cleaved caspase-3 (*p* = 0.008, one-way ANOVA), to a similar level as FAC (Fig. 6*A*). Interestingly, cholesterol-MβCD also induced expression of the shorter TNFR1 isoform (*p* = 0.002, one-way ANOVA) (Fig. 6*B*).

### Modulation of cholesterol metabolism rescues iron-dependent apoptosis

**Figure 7:**
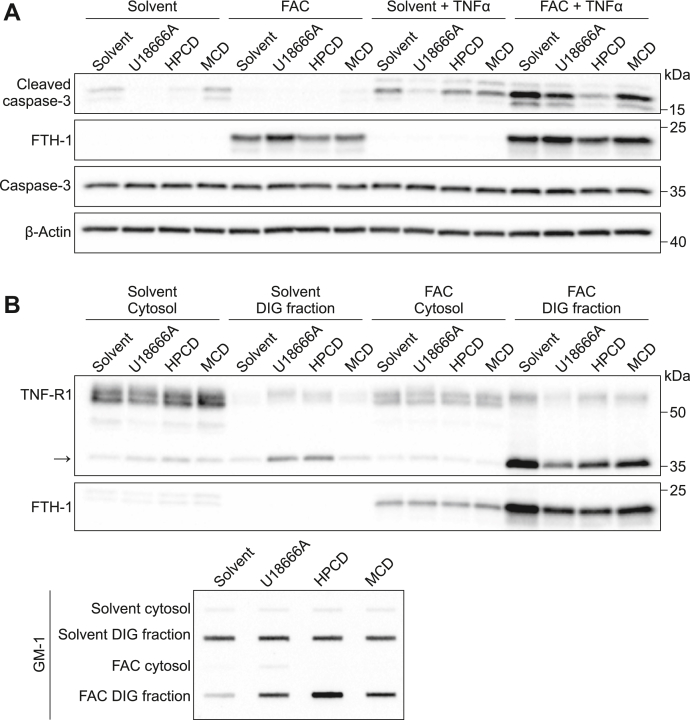
**Cholesterol depletion rescues apoptosis of HUVECs induced by excess iron independently of TNFR1 short isoform.***A*, HUVECs were treated with solvent (DMSO) or pharmacologically depleted of cholesterol by treating with 1 μM U18666A, 0.3% HPCD, or 50 μM MCD in the presence of 100 μM FAC for 24 h before stimulation with 50 ng/ml TNFα for 16 h. Western blot for cleaved caspase-3. β-Actin was used as a loading control. *B*, HUVECs were treated with solvent (DMSO), 1 μM U18666A, 0.3% HPCD, or 50 μM MCD in the presence or absence of 100 μM FAC for 40 h. Detergent soluble (cytosol) and insoluble glycosphingolipid (DIG) fractions were isolated by centrifugation. Proteins from equal volume of collected fractions were separated by SDS-PAGE and analyzed by Western blotting. To analyze the distribution of GM-1, each fraction was dot-blotted onto a nitrocellulose membrane and detected using CTx^HRP^. FAC, ferric ammonium citrate; HPCD, (2-hydroxypropyl)-β-cyclodextrin; HUVECs, human umbilical vein endothelial cells; MCD, methyl-β-cyclodextrin; TNFα, tumor necrosis factor-α.

To evaluate the role of lipid metabolism in FAC-potentiated apoptosis, HUVECs were treated with different drugs that alter cellular cholesterol content and then treated with 100 μM FAC and 50 ng/ml TNFα. U18666A (1 μM) was used to inhibit cholesterol movement out of lysosomes, whereas (2-hydroxypropyl)-β-cyclodextrin (HPCD, 0.3%) and methyl-β-cyclodextrin (MCD, 50 μM) were used to deplete cholesterol. As expected, FAC strongly potentiated cleaved caspase-3 expression induced by TNFα (Fig. 7*A*). Treatment of FAC-loaded cells with neither U18666A nor MCD was sufficient to rescue apoptosis (Fig. 7*A*). However, treatment with HPCD was protective against FAC-potentiated apoptosis (Fig. 7*A*). Although MCD and HPCD are both reported to remove membrane cholesterol, it is likely that the MCD dose we used (50 μM) was too low to reduce cholesterol levels, as cholesterol depletion was previously reported with 50- to 600-fold higher concentrations (25, 26). Using lipid raft isolation, we evaluated the changes in TNFR1 isoforms after pharmacological lowering of cellular cholesterol. As expected, TNFR1 short isoform expression was strongly potentiated by FAC in the lipid raft fractions; however, U18666A, MCD, nor HPCD altered TNFR1 expression (Fig. 7*B*). Considering that apoptosis was rescued with HPCD despite prominent TNFR1 short isoform expression, our data suggest that altered lipid metabolism rather than TNFR1 isoform expression is the adverse mediator of iron-potentiated apoptosis.

Taken together, our data suggest that iron loading of endothelial cells alters lipid metabolism, which sensitizes cells to apoptotic death by TNFα. Iron as well as cholesterol loading also induces accumulation of a short isoform of TNFR1. Although the accumulation of the short TNFR1 isoform is not sufficient to induce apoptosis, it remains to be determined whether it is needed in conjunction with changes in cholesterol metabolism to induce this new form of iron-dependent cell death.

## Discussion

NTBI appears in circulation in diseases of iron overload when the iron-binding capacity of transferrin is exceeded. Utilizing the ZIP14 transporter, the liver rapidly clears NTBI from plasma to store iron in hepatocytes, but with chronic exposure to NTBI, the liver becomes iron-overloaded, resulting in an increased risk for hepatic fibrosis, cirrhosis, and hepatocellular carcinoma (27). Apart from hepatocytes, several other cell types including cardiomyocytes, pancreatic cells, and pituitary cells can take up NTBI and become iron-overloaded, with consequent organ dysfunction. Before reaching any of those cell types, however, NTBI will first contact endothelial cells, but how excess iron affects endothelial cells has not been explored. We used a primary endothelial cell culture model to mimic the exposure of endothelial cells to NTBI in diseases with systemic iron overload. Using RNA-Seq to determine how endothelial cells respond to excess iron, we first found that excess iron in HUVECs induces cholesterol biosynthesis.

Cholesterol has essential functions in mammalian systems and its availability is regulated through homeostatic mechanisms to prevent the pathologic consequences of deficient or excessive cholesterol (28). The SREBPs are transcription factors that have well-defined roles in lipid homeostasis (29). Three SREBP isoforms, SREBP-1a, -1c, and -2, have been identified in mammals. SREBP-1a controls both cholesterol and fatty acid biosynthetic pathways by potently activating all SREBP-responsive genes. SREBP1c preferentially regulates transcription of genes involved in fatty acid synthesis (30), whereas SREBP2 preferentially activates genes involved in cholesterol synthesis (31), although a moderate induction in genes involved in fatty acid synthesis also occurs (32). In our study, we found that iron loading induced mRNA expression of *SREBP1a*, *SREBP1c*, and *SREBP2*, their gene targets, as well as synthesis of palmitoleic acid and cholesterol, indicating a broader role of iron in modulating lipid homeostasis.

The pathophysiologic role of iron-mediated changes in lipid homeostasis is uncertain. We considered whether this mechanism was triggered as a response to membrane damage from iron-induced prooxidants, but no differences were detected in oxidation of membrane phospholipids after 40-h exposure to iron. It is possible that the changes in lipid metabolism induced by iron facilitate membrane repair that prevents damage during chronic iron exposure. Our finding highlights a potential mechanism by which excess iron contributes to the development of pathology in iron disorders and chronic inflammatory diseases, which are exacerbated by iron overload.

The association of high iron status with altered cholesterol metabolism is increasingly recognized, although the data are occasionally conflicting. An early study in rats correlated hepatic iron content with circulating cholesterol levels, although hepatic iron retention was induced by dietary copper deficiency rather than dietary iron loading (33). A more direct comparison was made in dietary iron-loaded rats, where hepatic iron loading correlated with circulating cholesterol, but reduced expression of genes involved in sterol synthesis (34). In contrast, in mice with dietary iron overload, hepatic iron content positively correlated with hepatic cholesterol content, but not circulating cholesterol levels, and positively correlated with genes involved in cholesterol biosynthesis (35). Similarly, dietary iron– and iron–dextran–loaded mice had increased mRNA and enzyme activity of stearoyl-CoA desaturase-1, an enzyme involved in fatty acid metabolism (36). In agreement, we also detected changes in lipid homeostasis in mouse livers after iron perturbation. We had access to microarray data from a published cohort of transferrin receptor 2 (TFR2)-mutant mice susceptible to spontaneous iron overload. The mice were iron-depleted (using iron-deficient diet in combination with phlebotomy) and refed for 1 or 21 days with an iron-sufficient diet (37). Microarray analysis of the liver mRNA showed an increase in cholesterol and fatty acid biosynthesis genes with iron repletion. Specifically, the microarray MOE 430 2.0 array (Affymetrix) included 25,915 unique genes, of which 2254 were annotated as belonging to a biochemical pathway. Out of the annotated pathway genes, 44 increased robustly with iron refeeding, and of those, eight belonged to the cholesterol synthesis pathway (*p* < 0.000001). Further studies in iron-loaded mouse models that utilize cell sorting or single-cell RNA-Seq are needed to determine which cell types increase cholesterol biosynthesis *in vivo*. In our *in vitro* study, we saw a strong induction of cholesterol biosynthesis genes by iron loading in HUVECs, but not in the hepatic cell line Hep3B, suggesting that endothelial cells rather than hepatocytes may be responsible for the changes observed in rodent models. However, the immortalized endothelial cell line teloHAEC did not increase cholesterol synthesis in response to iron treatment, which indicates this response may not be universal to endothelial cells or the required machinery is lost with immortalization. Primary HUVECs were chosen based on their widespread use in vascular studies and their commercial availability. It is important to note that the HUVECs better represent a model of large vessel endothelial cells rather than microvascular endothelial cells, and it remains to be determined whether iron similarly perturbs cholesterol metabolism in primary endothelial cells from other tissues or other immortalized endothelial cell lines.

Importantly, we showed that iron interacts with the TNFα pathway. Excess iron worsened TNFα-induced apoptosis, as evidenced by increased levels of cleaved caspase-3. Iron loading further affected the TNFα pathway by inducing novel proteolytic processing of TNFR1 to promote accumulation of a shorter TNFR1 isoform in lipid rafts. In agreement, aberrations in cholesterol-rich lipid rafts are shown to promote apoptosis through TNFR1 (22). Similar to the adverse effects of iron, we noted accumulation of the short TNFR1 isoform after cholesterol treatment of HUVECs and increased cleaved caspase-3 after cotreatment of HUVECs with cholesterol and TNFα. Examining the interaction of cholesterol and excess iron in HUVECs, we determined that cholesterol depletion was sufficient to rescue iron-potentiated apoptosis, but this occurred even in the presence of the shorter TNFR1 isoform. Interestingly, Hep3Bs and teloHAECs did not show altered susceptibility to apoptosis or cholesterol homeostasis with FAC loading but did demonstrate conservation of the TNFR1 processing mechanism. This implies that the generation of the short TNFR1 isoform is not sufficient to induce apoptosis. We speculate that susceptibility to iron-potentiated apoptosis requires both induction of cholesterol biosynthesis and alterations in the TNFR1 pathway manifested as processing of the short TNFR1 isoform, with endothelial cells being particularly sensitive to excess iron. Although there is a large body of evidence demonstrating that disease susceptibility and response to infection worsens with elevated iron stores (38, 39, 40), any interaction between iron and cytokine-driven apoptosis has not previously been reported. Future studies are needed to determine the contribution of iron loading to endothelial apoptosis in inflammatory conditions *in vivo*, particularly those characterized by increased TNFα.

The adverse synergy between iron overload and inflammation could be important in multiple human diseases. In hereditary hemochromatosis, excess iron deposition in hepatocytes can result in progressive injury and fibrosis, which leads to production of proinflammatory and profibrogenic cytokines that further exacerbate hepatic injury (41). Elevations of proinflammatory cytokines TNFα and IL2 have been reported in iron-loaded thalassemic patients (42). Sickle cell disease, especially in its later stages, is likewise characterized by chronic inflammation as well as transfusional iron overload (43). In addition, biomarkers of oxidative stress are also increased in thalassemia, hemochromatosis, and sickle cell disease, which can further stimulate proinflammatory responses. There is also evidence that inflammatory chronic diseases are influenced by iron status. Milder excess iron has been linked to metabolic disorders including nonalcoholic fatty liver disease (44, 45, 46), diabetes (47, 48, 49, 50), and metabolic syndrome, in which iron stores correlate with markers of chronic inflammation (51, 52). Overall, the evidence suggests that iron overload worsens inflammatory diseases and that iron accumulation increases the risk for development of diseases with underlying inflammation.

Our observation of the interaction between iron overload and inflammation may not only apply to iron-loading conditions affected by chronic inflammation but may also be relevant during episodes of acute inflammation, such as infections. Furthermore, iron loading predisposes patients to severe infections with gram-negative pathogens (53). It remains to be determined whether the endothelium-targeting adverse synergy between iron and inflammation contributes to increased morbidity and mortality of iron-overloaded patients affected by severe infections.

Evidence of endothelial dysfunction has indeed been reported in iron overload disorders (54). Patients with hemochromatosis were reported to develop endothelial dysfunction, and therapeutic iron depletion was associated with improvement in endothelial function (55). Vasculopathy and endothelial dysfunction is one of the hallmarks of sickle cell disease pathophysiology (56) and is increasingly recognized in thalassemia as a contributor to disease-related complications (57, 58). Iron overload was also reported to worsen atherosclerosis in mice by promoting lipid peroxidation, endothelial dysfunction, and inflammation (59). In the same study, an inverse correlation between the severity of therapeutic iron depletion and circulating biomarkers of endothelial dysfunction and inflammation in a contemporary hemochromatosis patient cohort was also reported. Together, these data support the disease relevance of adverse interactions between iron and inflammation affecting endothelial function.

In this study, we demonstrate how NTBI accumulation affects cultured endothelial cells. Iron loading induces cholesterol biosynthesis, promotes novel TNFR1 proteolytic processing, and sensitizes cells to TNFα-mediated apoptosis. We determined that during conditions of iron overload, iron-driven changes in cholesterol homeostasis potentiate apoptosis driven by TNFα. Our findings have important implications for iron-loading conditions, especially when inflammation is present. Altered cholesterol metabolism by excess iron in endothelial cells may contribute to iron-mediated toxicity in human iron-overload disorders.

## Experimental procedures

### Cell culture

Primary HUVECs pooled from ten donors were obtained from the American Type Culture Collection (ATCC #PCS-100-013). HUVECs were cultured in complete endothelial cell growth media (Cell Applications #211-500) at 37 °C in a 5% CO_2_ and 95% air atmosphere. For experiments, HUVECs were plated on collagen and experiments were performed from passages 3 to 6.

### Reagents

Unless otherwise specified, all chemicals were obtained from Sigma-Aldrich. For iron-loading studies, HUVECs were treated with 100 μM FAC (MP Biomedicals #158040), 100 μM FAS (F-1543), 100 μM ferric chloride (#157740), 2 mg/ml apoferritin (#A-3641), 2 mg/ml holoferritin (#F-4503), 100 μM cupric chloride (#C-6917), or 100 μM zinc sulfate (Fisher #Z-58) for the indicated times.

Cholesterol-methylβcyclodextrin complexes were prepared as follows: a 5% w/v solution of MCD (#C4555) was prepared in water by heating to 70 °C, and 10 mg/ml cholesterol (#C8667) in 100% ethanol was added dropwise. Cholesterol-MβCD was stirred until the solution was clear, the solvent was evaporated overnight by speed-vac, and cholesterol-MβCD was reconstituted in MilliQ water to 2.5 mM, filtered, and stored at 4 °C. HUVECs were treated with 0 to 50 μM cholesterol-MβCD for 40 h. For cholesterol-depletion studies, HUVECs were treated with 1 μM U18666A (Cayman Chemicals #10009085), 0.3% HPCD (#H107), or 50 μM MCD for the indicated times. For inflammation studies, HUVECs were treated with 50 ng/ml recombinant human TNFα (BioLegend #570104) for 6 or 16 h. The optimal time of culture with TNFα was based on preliminary studies testing concentration and time dependence.

For protease inhibitor experiments, HUVECs were treated with 25 μM TAPI2 (Calbiochem, #579052), 1 μM DAPT (Calbiochem, #565770), or protease inhibitors (from Sigma #INHIB1) 6-aminohexanoic acid (E-ACA, 40 mM), antipain (100 μM), benzamidine HCl (4 mM), aprotinin (1 μM), bestatin (40 μM), chymostatin (100 μM), E-64 (10 μM), EDTA (1 mM), leupeptin (100 μM), pepstatin A (1 μM), phosphoramidon (10 μM), soybean trypsin inhibitor (STI, 5 μM), or protease inhibitor cocktail (1:200, Sigma P1860) for 24 h.

The specific time of culture with FAC for cholesterol biosynthesis analysis, apoptosis, and TNFR1 processing was based on preliminary time-course experiments to determine iron loading of HUVECs and their susceptibility to apoptosis (see Fig. 4*B* as an example).

### RNA sequencing

RNA sequencing was performed by the UCLA Technology Center for Genomics and Bioinformatics Core Facility. Libraries for RNA-seq were prepared with the KAPA Stranded kit, and data were sequenced on Illumina HiSeq 3000 for a single-read 50-bp run. Data quality check was done on Illumina SAV. Demultiplexing was performed with Illumina Bcl2fastq2 v 2.17 program. Total RNA from primary HUVECs was extracted using an RNeasy Micro kit (QIAGEN) following the manufacturer’s instructions. The reads were mapped to the latest UCSC transcript set using Bowtie 2 version 2.1.0, and the gene expression level was estimated using RSEM v1.2.15. The trimmed mean of M-values was used to normalize gene expression. Differentially expressed genes were identified using the edgeR program. Expression data were analyzed by comparing control cells treated with solvent (N = 3) with cells treated with 100 μM FAC (N = 3) for 30 h. Genes identified by RNA sequencing were validated by quantitative real-time PCR. The RNA-Seq data have been deposited in NCBI’s Gene Expression Omnibus and are accessible through the GEO Series accession number GSE168534 (https://www.ncbi.nlm.nih.gov/geo/query/acc.cgi?acc=GSE168534).

### Gene expression quantification by RT-PCR

HUVECs were lysed in TRIzol Reagent (Life Technologies), and total RNA was isolated by chloroform extraction. Five hundred nanograms of RNA was reverse-transcribed using the iScript cDNA Synthesis Kit (Bio-Rad). Quantitative real-time PCR was performed on cDNA using SsoAdvanced SYBR Green Supermix (Bio-Rad) on the CFX Real-Time PCR Detection System (Bio-Rad). Samples were measured in duplicate, and target genes were normalized to *HPRT*. Data are expressed as 2^−ΔΔCt^. Primer sequences are provided in Table 1.

**Table 1:** List of human primers for qRT-PCR

Gene name	Primer sequences
*HPRT*	Forward: 5′- GCC CTG GCG TCG TG ATTA GT -3′ Reverse: 5′- AGC AAG ACG TTC AGT CCT GTC -3′
*SREBP2*	Forward: 5′- AGG AGA ACA TGG TGC TGA -3′ Reverse: 5′- TAA AGG AGA GGC ACA GGA -3′
*MVD*	Forward: 5′- ATC AAG TAC TGG GGC AAG CG -3′ Reverse: 5′- TTC AGC CAA ATC CGG TCC TC -3′
*LDLR*	Forward: 5′- GGG CTC TGT CCA TTG TCC TC -3′ Reverse: 5′- ACC ATC TGT CTC GAG GGG TAG -3′
*HMGCR*	Forward: 5′- TTC GGT GGC CTC TAG TGA GAT -3′ Reverse: 5′- GTC ACT GCT CAA AAC ATC CTC TTC -3′
*TFRC*	Forward: 5′- AGT TGA ACA AAG TGG CAC GAG -3′ Reverse: 5′- AGC AGT TGG CTG TTG TAC CTC -3′
*TNFRSF1A*	Forward: 5′- CGC TAC CAA CGG TGG AAG TC -3′ Reverse: 5′- CAA GCT CCC CCT CTT TTT CAG -3′
*SREBP1a*	Forward: 5′- TGC TGA CCG ACA TCG AAG AC -3′ Reverse: 5′- CCA GCA TAG GGT GGG TCA AA -3′
*SREBP1c*	Forward: 5′- CCA TGG ATT GCA CTT TCG AA -3′ Reverse: 5′- CCA GCA TAG GGT GGG TCA AA -3′
*ACCA*	Forward: 5′- CTG TAG AAA CCC GGA CAG TAG AAC -3′ Reverse: 5′- GGT CAG CAT ACA TCT CCA TGT G -3′
*FASN*	Forward: 5′- TCG TGG GCT ACA GCA TGG T -3′ Reverse: 5′- GCC CTC TGA AGT CGA AGA AGA A -3′

### Lipid analysis

Lipid compositional analysis was performed by the UCLA Lipidomics Core Facility. For stable isotope labeling, HUVECs were cultured in endothelial cell growth media supplemented with 5 mM [U^13^C]-D-glucose with and without 100 μM FAC for 30 or 40 h to assess the time course of cholesterol synthesis. Analysis of fatty acid and cholesterol synthesis in normal and iron-loaded HUVECs was performed on an Agilent 7890B/5977A GC-MS instrument. Data are presented as nanomole synthesis/million cells and was estimated using isotopic spectral analysis.

Oxidized phospholipids were extracted from HUVECs treated with 100 μM FAC for 30 h, to determine whether membrane damage precedes cholesterol synthesis, using a biphasic butanol extraction. Cells were washed in PBS and collected in 1-butanol and transferred to a glass tube. NaCl (10%) was added to each tube and centrifuged at 2000*g* for 10 min at room temperature (RT). The upper organic phase was evaporated under argon gas in a 37 °C water bath and contents were solubilized in 150 μl methanol. Sample was centrifuged, and the clear supernatant was stored at −80 °C until analysis. Oxidized phospholipids were measured by LC-MS using an internal oxidized palmitoyl arachidonoyl phosphatidylcholine standard.

### Lipid raft isolation

Lipid rafts were isolated using discontinuous sucrose gradient centrifugation as previously described (60). Two 150-mm dishes of cultured HUVECs were combined for each treatment group. Detergent-insoluble membrane fractionation was performed as described (61), using HUVECs cultured on 150 mm dishes. Equal volumes of proteins from each fraction were resolved by SDS-PAGE. To identify the lipid raft and detergent-insoluble membrane fractions, equal volumes of proteins were dot-blotted onto nitrocellulose and probed for ganglioside GM1.

### Western blotting

HUVECs were lysed by mechanical disruption in the RIPA lysis buffer with freshly added protease inhibitors (Santa Cruz #SC-24948). Cell lysates were centrifuged at 21,000*g* for 15 min at 4 °C, and protein concentration was measured by the bicinchoninic acid assay using bovine serum albumin as a standard. Proteins (20 μg/lane) were separated by SDS-PAGE and transferred to nitrocellulose membranes. Membranes were blocked for 1 h in 5% w/v dried nonfat milk or bovine serum albumin in TBS with 0.1% Tween-20 and incubated with primary antibodies in the blocking buffer overnight at 4 °C: cleaved caspase 3 (rabbit, 1:2000, Cell Signaling Technology #9664), total caspase 3 (rabbit, 1:5000, Cell Signaling Technology #9662), ferritin light chain (goat, 1:5000, Novus Biologicals #NBP1-06986), ferritin heavy chain 1 (rabbit, 1:25,000, Cell Signaling Technology #4393), TNFR1 (rabbit, 1:5000, Cell Signaling Technology #3736), Cholera Toxin B subunit peroxidase conjugate (1:10,000, Sigma #C3741), and β-actin-peroxidase (1:50,000, Sigma A3854). The secondary reaction was performed using HRP-conjugated anti-rabbit or anti-goat IgG diluted 1:5000 in the blocking buffer. Protein blots were visualized by chemiluminescence using the ChemiDoc XRS+ imaging system and quantified using Image Lab software (Bio-Rad). Antibodies were validated based on the expected molecular weight, use of controls, or by knocking down the proteins of interest using siRNA.

### Filipin staining

Filipin staining was performed using a cholesterol cell-based assay kit following the manufacturer’s instructions (Cayman Chemicals 10009779). SYTOX green was used as a nuclear counterstain. Images were captured using a Zeiss LSM700 confocal microscope. Grayscale confocal fluorescence images were pseudo-colored with turquoise to represent the cholesterol and violet to indicate the nucleus.

### Lentivirus generation

Hek293t cells were grown to 70% confluence in 150 mm poly-D-lysine–coated plates in 10% fetal calf serum with antibiotics. Hek293t cells were transfected with 2 μg C-terminal V5-tagged TNFR1 pLX304 (DNasu #HsCD00438626), 5 μg pMD2.6 (Addgene #12259), 7.5 μg pMDLg/pRRE (Addgene #12251), and 7.5 μg pRSV-REV (Addgene #12253) using Lipofectamine 2000 (Thermo Fisher) for 16 h. The supernatant was replaced with fresh DMEM with 10% fetal calf serum. Viral supernatant was harvested after 24 and 48 h, pooled, and centrifuged at 300*g* for 5 min to pellet cells. The supernatant was filtered through a 0.45-μm low protein binding filter and stored at 4 °C until plasmid digestion. To digest plasmid DNA, the supernatant was treated with DNase-1 (Sigma D4527, 10 U per 10 μg of total plasmid DNA) at RT for 30 min followed by 4 h at 4 °C. Virus was concentrated by ultracentrifugation at 70,000*g* for 2 h at 12 °C. Viral pellets resuspended in Hank's balanced salt solution were layered on 20% sucrose and centrifuged at 50,000*g* for 2 h at 12 °C. The supernatant was snap-frozen in liquid nitrogen and stored at −80 °C.

For transduction experiments, HUVECs were treated with increasing volume of lentivirus for 10 h before incubation with solvent or 100 μM FAC for 24 h. Cell lysates were resolved by SDS-PAGE and probed for the transduced V5-tag.

### Statistical analysis

Data are presented as box and whisker plots, where the box plot indicates the upper 75th and lower 25th percentiles and the whiskers indicate the 90th and 10th percentiles. Within the box, the solid line indicates the median and the dotted line the mean. Statistical analysis was performed using SigmaPlot, version 12.5 (SYSTAT Software). Principal component analysis was performed using R and Transcripts Per kilobase Million values. Statistical differences between groups were determined by one-way ANOVA followed by Holm–Sidak method for multiple comparisons for normally distributed values, one-way ANOVA on ranks followed by Dunn’s method for multiple comparisons of non-normally distributed values, two-tailed Student’s *t* test for normally distributed values, or Mann–Whitney *U* test for non-normally distributed values. Statistical test, number of biological replicates, and *p*-value are indicated in each figure panel. A *p*-value of <0.05 was considered significant.

## Data availability

All data are contained within the article and its supporting information. RNA-Seq data have been deposited in NCBI’s Gene Expression Omnibus and are accessible through the GEO Series accession number GSE168534 (https://www.ncbi.nlm.nih.gov/geo/query/acc.cgi?acc=GSE168534).

## Supporting information

This article contains supporting information.

## Conflict of interest

T. G. and E. N. are shareholders and scientific advisors of Intrinsic LifeSciences and Silarus Therapeutics, and consultants for Ionis Pharmaceuticals, Protagonist, Disc Medicine, and Vifor. T. G. is a consultant for Akebia. Other authors declare that they have no conflicts of interest with the contents of this article.
