# Supplementary material for: Iron loading induces cholesterol synthesis and sensitizes endothelial cells to TNFα-mediated apoptosis
Source: J Biol Chem. 2021 Sep 2;297(4):101156. doi: 10.1016/j.jbc.2021.101156 (PMC8463868; doi:10.1016/j.jbc.2021.101156)
Supplement: Supplemental Figures S1–S8 [file mmc1.pdf]

SUPPORTING INFORMATION FOR:

Iron loading induces cholesterol synthesis and sensitizes endothelial cells to TNFα-mediated apoptosis

Allison L Fisher[1,3], Daniel N Srole[2,3], Nicolaos J Palaskas[3], David Meriwether[4], Srinivasa T Reddy[4], Tomas Ganz[3], Elizabeta Nemeth[3]*

LIST OF MATERIALS INCLUDED:

1. Supplemental figures 1-8
2. Supplemental materials & methods

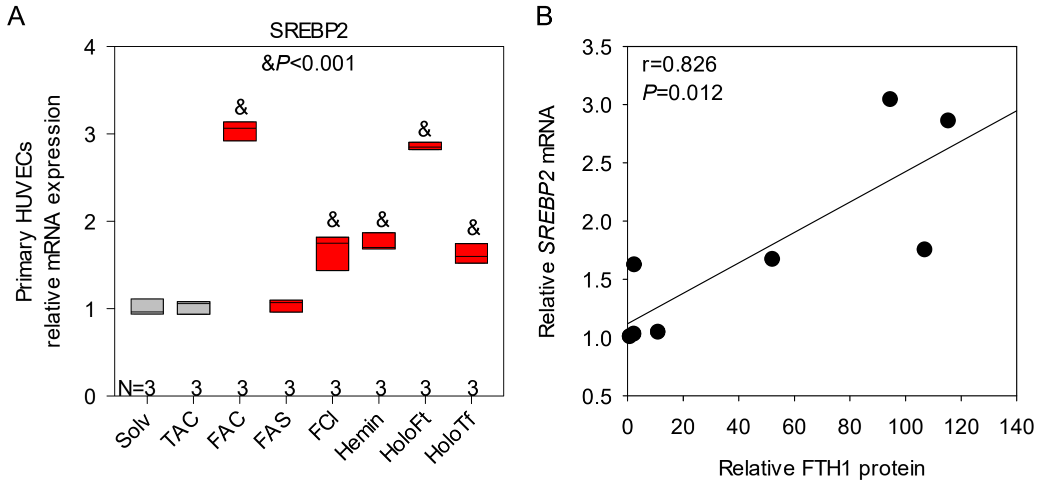

Supplemental Figure 1. Cellular iron loading induces *SREBP2* mRNA expression. Primary HUVECs were treated with solvent (water) or triammonium citrate (TAC, 100 µM) (grey) or different forms of iron (red) including ferric ammonium citrate (FAC, 100 µM), ferric ammonium sulfate (FAS, 100 µM), ferric chloride (FCl, 100 µM), hemin chloride (20 µM), holo-ferritin (Ft, 2 mg/ml) or holo-transferrin (Tf, 100 µM) for 40 h. N=3 biological replicates per condition. **(A)** *SREBP2* mRNA expression relative to solvent. Data are expressed as $2^{-\Delta\Delta Ct}$. **(B)** Pearson correlation between *SREBP2* mRNA and ferritin heavy chain 1 (FTH1) protein in HUVECs. Statistical differences between groups were determined by one-way ANOVA with Holm-Sidak method for multiple comparisons for normally distributed values (denoted by &).

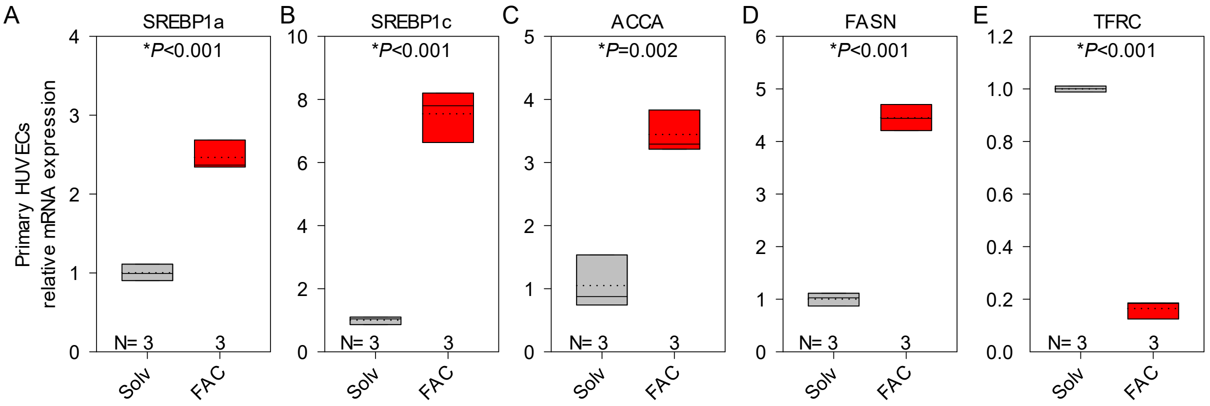

Supplemental Figure 2. Iron induces SREBP-1 target genes in HUVECs. Primary HUVECs were treated with solvent (water, grey) or 100 µM ferric ammonium citrate (FAC, red) for 40 h. **(A-E)** qPCR analysis of fatty acid biosynthesis genes *SREBP1a*, *SREBP1c*, *ACCA*, *FASN*, and iron importer *TFRC*. Data are expressed as $2^{-\Delta\Delta Ct}$. Number of biological replicates are indicated above the *x*-axis. Statistical differences between groups were determined by Student's *t*-test for normally distributed values (denoted by *).

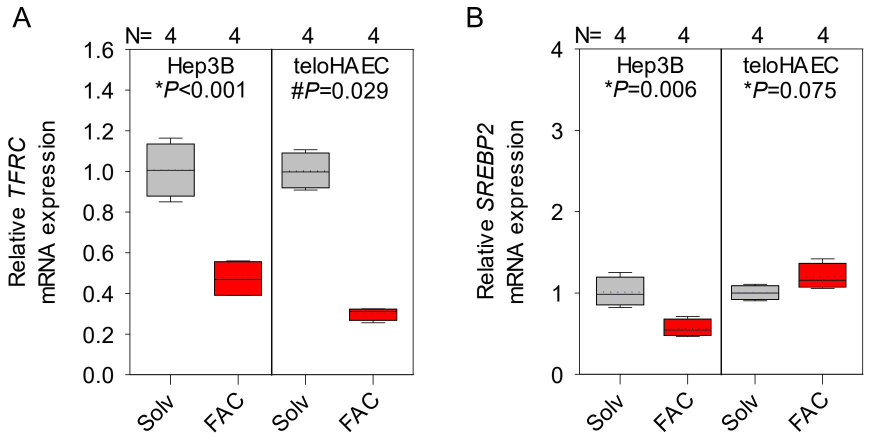

Supplemental Figure 3. Cell panel for induction of *SREBP2* by iron loading. Human hepatic cell line Hep3B and immortalized endothelial cells teloHAEC were treated with solvent (water, grey) or 100 µM FAC (red) for 40 h. **(A)** *TFRC* and **(B)** *SREBP2* mRNA expression was determined by qRT-PCR and data are shown as $2^{-\Delta\Delta Ct}$. Number of replicates are indicated above the *x*-axis. Statistical differences between groups were determined by Student's *t*-test for normally distributed values (denoted by *) or Mann-Whitney *U* for non-normally distributed values (denoted by #).

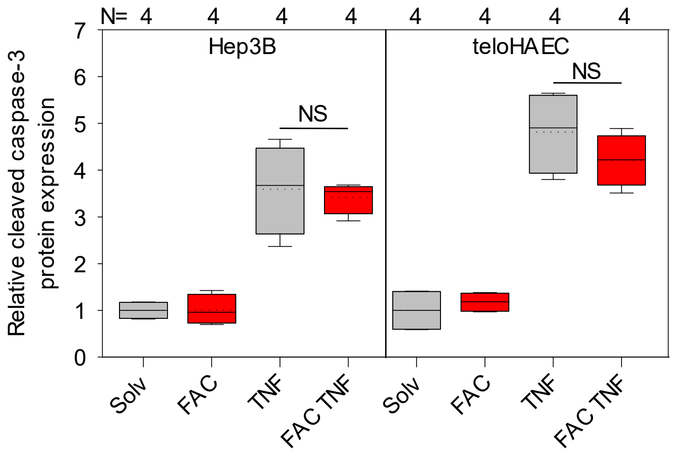

Supplemental Figure 4. Cell panel for potentiation of TNFα-induced apoptosis by iron loading. Human cell lines Hep3B and immortalized endothelial teloHAECs were treated with solvent (water, grey) or 100 µM FAC (red) for 24 h following stimulation with 50 ng/ml TNFα for 16 h in normal or FAC-supplemented media. Cleaved caspase-3 protein expression was determined by Western blotting and normalized to β-actin. Number of replicates are indicated above the *x*-axis. Statistical differences between groups were determined by one-way ANOVA.

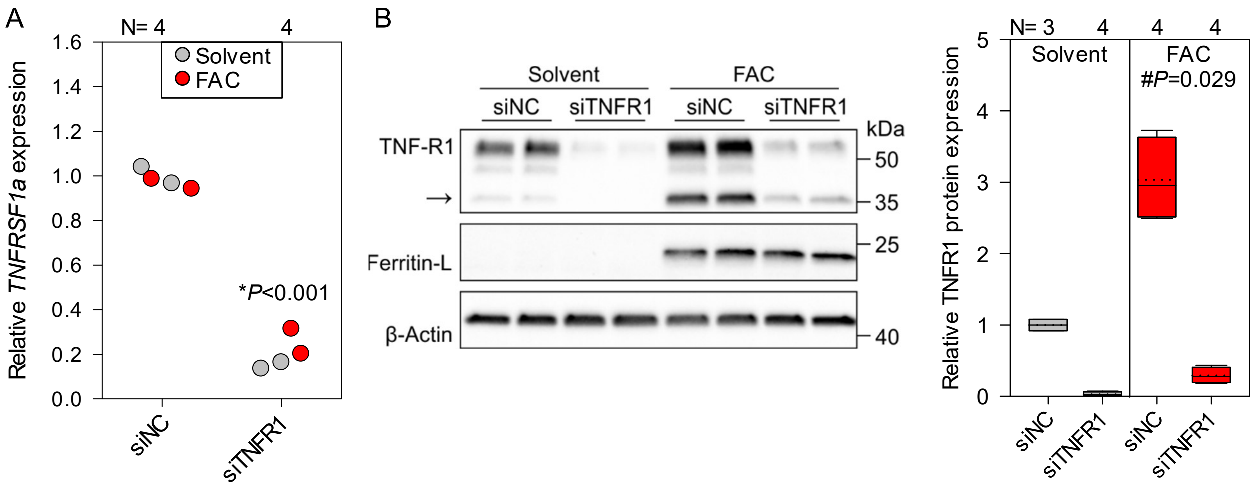

Supplemental Figure 5. Validation of TNFR1 antibody in HUVECs. HUVECs were reverse transfected with 12.5 pmol negative control siRNA or siRNA targeting TNFR1 for 48 h and treated with solvent (water, grey) or 100 µM FAC (red) for 24h. **(A)** *TNFRSF1A* mRNA expressed as $2^{-\Delta\Delta Ct}$ and **(B)** TNFR1 protein expression by Western blotting. β-actin was used as a loading control. Representative image from N=4 independent experiments. Number of replicates are indicated above the figure panels. Statistical differences between groups were determined by Student's *t*-test for normally distributed values (denoted by *) or Mann-Whitney *U* for non-normally distributed values (denoted by #).

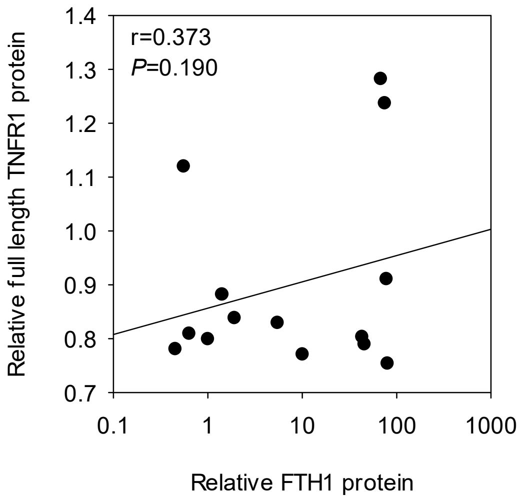

Supplemental Figure 6. Full length TNFR1 does not correlate with cellular iron loading in HUVECs. HUVECs were treated with solvent (water) or 100 µM FAC, ferric ammonium sulfate (FAS, 100 µM), ferric chloride ($FeCl_3$, 100 µM), apo-ferritin (FT, 2 mg/ml), holo-FT (2 mg/ml), copper chloride ($CuCl_2$, 100 µM), or zinc sulfate ($ZnSO_4$, 100 µM) for 24 h. Pearson correlation between ferritin heavy chain (FTH1) and TNFR1 protein by Western blotting and normalized to β-actin.

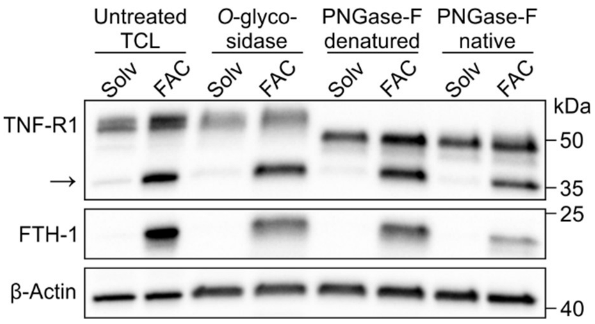

Supplemental Figure 7. TNFR1 glycosylation in HUVECs. HUVECs were treated with water solvent or 100 µM FAC for 30 h. Western blot for full-length and short TNFR1 isoform (indicated by arrow) of solvent and FAC-treated lysates after incubation with or without *O*-glycosidase or PNGase F. β-actin was used as a loading control.

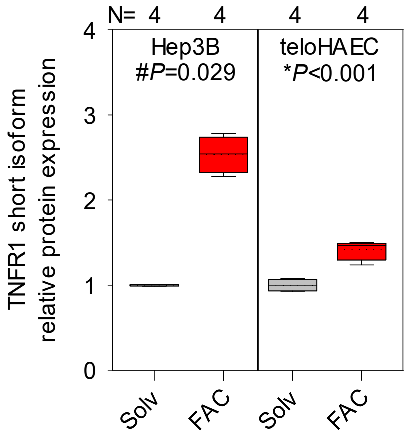

Supplemental Figure 8. Induction of a short isoform of TNFR1 with iron loading in different cell lines. Human hepatic cell line Hep3B and immortalized endothelial teloHAECs were treated with solvent (water, grey) or 100 μM FAC (red) for 40 h. TNFR1 short isoform protein levels were determined by Western blotting and normalized to β-actin. Number of replicates are indicated above the *x*-axis. Statistical differences between groups were determined by Student's *t*-test for normally distributed values (denoted by *) or Mann-Whitney *U* for non-normally distributed values (denoted by #).

Supplemental materials & methods

Cell culture

HUVECs were cultured in complete endothelial cell growth media (Cell Applications #211-500). Hep3B cells were cultured in DMEM supplemented with 10% FBS. TeloHAECs were cultured in complete MCDB-131 media (VEC Technologies). Cells were cultured at 37°C in a 5% CO_2 95% air atmosphere.

Reagents

Unless otherwise specified, all chemicals were obtained from Sigma-Aldrich. For the indicated times, cells were treated with ferric ammonium citrate (FAC, 100 μM), ferrous ammonium sulfate (100 μM), ferric chloride (100 μM), hemin chloride (20 μM, #H-1652), holoferritin (2 mg/ml), holotransferrin (100 μM, Serologicals Corporation #4455-01), or triammonium citrate (100 μM , #A1332) as a control. For inflammation experiments, cells were treated with 50 ng/ml recombinant human TNFα (Biolegend #570104) for the indicated times.

siRNA knockdown of TNFR1

SMARTPool siRNA targeting human TNFR1 (Dharmacon SMARTPool L-005197-00-0005) was used to suppress *TNFRSF1A* expression. Non-targeting siRNA was used as a control (Dharmacon D-001810-10-05). Reverse transfection was performed using Lipofectamine RNAiMAX (ThermoFisher) in Opti-MEM media following the manufacturer's protocol to yield a final concentration of 12.5 pmol siRNA per well. HUVECs were reverse transfected for 48 h and treated with 100 μM FAC for 24 h. Knockdown was confirmed by qRT-PCR and immunoblotting.

TNFR1 deglycosylation

HUVECs were treated with 100 μM FAC for 30 h. Cell lysates were digested with *O*-glycosidase to cleave *O*-glycans or PNGase F to cleave *N*-linked glycans following the manufacturer's protocol (New England Biolabs #E0540S). *N*-linked deglycoslyation was performed under denaturing and non-denaturing conditions.
